# Description of the New Hospital Wings of the Royal Asylum, Perth
*"Description of the New Hospital Wings of the Royal Asylum, Perth. By R. Urquart, M.D., and A. Heiton, F.R.I.B.A. (Pamphlet with Plans Reprinted from the Journal of Mental Science.)


**Published:** 1890-04-26

**Authors:** 


### ASYLUM CONSTRUCTION.

### DESCRIPTION OF THE NEW HOSPITAL WINGS OF THE ROYAL ASYLUM, PERTH.*

IN examining these plans we experienced the same difficulty we have met on former occasions, namely, the difficulty in the absence of a contour plan of the ground of fairly judging whether the additions have been carried out in the best manner, as without such plan, or without knowing what space was available for building operations, it is impossible to criticise the position of the new work. At first sight we were of opinion that the dormitory and day-room parts of the new hospital wings under notice should have been placed on the male side more to the west, and on the female side more to the east than they are, and thus have obtained a full southern exposure, which in a cold climate means a great deal to the comfort of the inmates. It is, of course, quite possible, and even probable, that this could not be done at the Perth Asylum.

Taking the blocks as they are, we find that the east end of each is completely taken up by the bath-room, w.c.'s, and the attendant's room. The latter are placed side by side, which can hardly be a pleasant arrangement for the attendant. The west ends are occupied by two cubicals, and the space between these forms a bay, which is very well lighted by a large, apparently, mullioned window. This seems a very good idea, and, as Dr. Urquhart remarks, some of the patients can get out of "the current of the general life in the rest of the room"; that is, of the day-room, of which the bay forms part. Another bay projects into this room, and is used for serving the food. The dormitory adjoins the day-room, and is of about the same size. It is difficult to see why the partition between these rooms was not constructed of glass and wood. There is no attendant's room overlooking this dormitory. There are three windows in the dormitory, one of which looks south and two north, so that a cross current of air is provided for. The day-room has only one window, irrespective of the bay.

The hospital ward may be approached either through a glass corridor or through the gallery for acute cases, to which it is joined by a corridor running north and south, having small bed-rooms on the east side, and a large bay on the west. Altogether, it may be inferred that the hospital ward will have a cheerful, homely appearance.

The heating is effected partly by open fire-places and partly by steam pipes. The ventilation is well carried out, and the system has been subjected to crucial tests. The main drain would seem to pass under the building, which is not a course to be commended; but here, again, there might have been no alternative.

The windows are well constructed, and are entirely free from the old asylum and prison look. The hinged fanlights are always useful for purposes of ventilation.

* "Description of the New Hospital Wings of the Royal Asylum, Perth. By R. Urquart, M.D., and A. Heiton, F.R.I.B.A. (Pamphlet with Plans Reprinted from the *Journal of Mental Science*.)

